# Plant-Derived Insecticides Under Meta-Analyses: Status, Biases, and Knowledge Gaps

**DOI:** 10.3390/insects11080532

**Published:** 2020-08-14

**Authors:** Leonardo M. Turchen, Lírio Cosme-Júnior, Raul Narciso C. Guedes

**Affiliations:** Departamento de Entomologia, Universidade Federal de Viçosa, Viçosa MG 36570-900, Brazil; leonardo.turchen@ufv.br (L.M.T.); lirio.junior@ufv.br (L.C.-J.)

**Keywords:** botanical insecticides, biorational insecticides, biopesticides, sublethal toxicity, non-target species, pest management

## Abstract

**Simple Summary:**

Botanical insecticides have a burgeoning body of literature over the last decades, which potentially hides misconceptions and limitations in this research field. Here, we conducted a systematic review to recognize the current context of the field, including the temporal trends, the main botanical families, and the taxonomic groups explored in papers between 1945-2019. Next, we performed meta-analyses to identify any existing biases on such studies and realized existing knowledge gaps worthy of attention. The 74 years of our review tracked over 2500 scientific papers, which exhibit an exponential growth since the 1990s, always with prevalent interest on Meliaceae plant species, and a dozen additional families, albeit 190 botanical families were investigated. These articles primarily focused on pest insects with rather little attention devoted to non-target species. Besides, they concentrate on mortality assessments among arthropod species, overlooking sublethal assessments. Such outcomes highlight that sublethal effects remain poorly understood in both target and non-target organisms signaling a relevant gap to be filled, which will lead to novel insights allowing potential applications in pest management.

**Abstract:**

Plant-derived or botanical insecticides are biopesticides experiencing substantial ongoing increase in interest. The 74 years of our literature survey tracked over 2500 papers on botanical insecticides published between 1945 and 2019 (Web of Science database). Such a survey allowed meta-analyses to recognize current status and biases of the studies providing important insights into the research topic. They include the recognition of the exponential growth of such studies since the 1990s, the prevalent interest on the Meliaceae plant species and a dozen additional families, although some 190 families have been investigated. The arthropods targeted by such studies were pest species (*ca*. 95%) with rather little attention devoted to non-target species (*p* < 0.001). This bias is followed by another one—mortality assessments are prevalent among target and non-target arthropod species when contrasted with sublethal assessments (*p* < 0.01). These omissions are pivotal, as they fail to recognize that sublethal effects may be as important or even more important than mortality, and that initial insecticide deposits quickly degrade over time leading to prevailing sublethal exposure. Furthermore, although the target of control is limited to few species, non-target species will be exposed and as such need to be factored into consideration. Thus, these biases in studies of botanical insecticides incur in knowledge gaps with potential consequences for the practical use of these compounds as pest management tools.

## 1. Introduction

The creation of the Garden of Eden probably did not involve the use of pesticides, nor any agrochemical for that matter. However, alas, it did not last as long as desired. A reversal on God´s inordinate fondness of beetles apparently took place, and insecticides were born. Nonetheless, there are controversies about this tale of pesticide genesis [1], what leads to another musing—if men had not procrastinated as much on the conception of insecticides, the Amorites would not have been defeated by the Israelites who were aided in their troubles by swarms of hornets, and lice and locust would not have plagued Egypt as much as to potentially compromise the Exodus. Thus, the history of the world would likely have been different. Nonetheless and regardless of their genesis, insecticides do exist and are ubiquitous permeating the Earth biosphere during the Anthropocene [2,3,4,5].

Insecticides are toxicants, and not necessarily toxins (i.e., poisonous substances from living cells or organisms), deliberately released into the environment. As their environmental presence takes place at higher levels than their natural background levels, insecticides are environmental contaminants. Furthermore, they adversely affect living species, justifying their recognition as pollutants [6]. This labeling should not detract the recognition of their importance in agriculture, animal husbandry, and public health [5,6,7]. Therefore, concepts, perceptions, and misconceptions should not cloud our understanding of the arguably most influential pest management tools since the onset of the late 1940s broad-scale use of insecticides. So, the neologisms, pleonasms, and misnomers that haunt this subject invite clarification for due understanding [6,8]. Within context, the insecticide paradox deserves attention since the human and environmental concerns with these compounds remain a counterpoint to their increasing worldwide use [7,9,10].

Insecticide is popularly defined as a substance that kills insects—emphasis on the “*killing*” aspect of the definition. Nonetheless, this concept is at odds with the technical definition of these compounds used within the scientific and regulatory framework as “any substance or mixture of substances intended for preventing, destroying, repelling, or mitigating any insect pest” (e.g., US Federal Insecticide, Fungicide, and Rodenticide Act). Thus, an initial divergence with perceptual bias and far-reaching consequences was recently recognized and explored within a conceptual framework of toxicology and ecotoxicology applied to pest management [6,11]. Another concern is the concept of biorational insecticides, or bioinsecticides for short, which are both associated with perceived advantages likely associated with the suffix “bio”. Both are promoted as suitable alternatives to synthetic insecticides in integrated pest management programs [6,8].

Biorational insecticides or bioinsecticides are generally defined as insecticides composed of natural products or derived from nature, including animals, plants, microbes, and minerals [8,12,13]. A few relevant positive attributes are usually associated with these compounds, including perceived low environmental and mammalian risk, higher specificity and safety to non-target organisms, lower risk of resistance development, and lower environmental persistence [14,15,16]. Although such perceptions hold true for some such compounds or even groups of compounds [8,17,18,19], generalizations tend to be deceitful [6,20,21,22,23]. Still, bioinsecticides represent a broad category of compounds and among them, plant-derived or botanical insecticides are at the forefront of interest, and popularity [24,25,26]. Botanicals represent the lion’s share of the interest in bioinsecticides contributing nearly half of the scientific publications dealing with bioinsecticides based on a recent review [8].

The myriad publications and ongoing interest on botanical insecticides potentially hide misconceptions and limitations [8,18]. The scenario invites some rethinking, as recently suggested for biopesticides in general [8]. The present effort is in sync with this call and concern, but is focused on botanical insecticides. With this in mind, a systematic literature survey on the subject was performed using the Web of Science database since its starting year 1945, until 2019. The data-gathering from the survey allowed meta-analyses of the data obtained from the studies with the objectives of (i) recognizing the magnitude of the evolution and focus of interest within the subject of botanical insecticides, (ii) identifying any existing biases on such studies, and (iii) realizing existing knowledge gaps worthy of attention.

Previous (qualitative) reviews recognize the surge in interest in botanical insecticides since the 1980s [8,18], leading us to suspect an even earlier interest and steeper increase in studies in more recent years. The multitude of contributions certainly hide biases, a trait prevalent in human nature as consequence of the perceptions and misperceptions from our species, but we want to learn the prevailing ones beyond the greater focus on a given plant species, like neem [18]. Therefore, (i) we performed a systematic literature survey to recognize the overall and temporal trends in the publications, considering the botanical family as moderators of variation. Subsequently, (ii) we performed meta-analyses to synthesize and test (a) whether the publications favored a particular target or non-target organism, and (b) whether the biological parameters have been recorded (or not) in papers with botanical insecticides. Such issues enable the overall understanding of the subject and allow recognition of sources of variation in outcomes, which is an essential component for the identification of subjects that are worth exploring. Thus, the present effort.

## 2. Materials and Methods

### 2.1. Data Collection

The process adopted for the systematic literature survey and subsequent meta-analyses followed the guidelines of “Preferred Reporting Items for Systematic Reviews and Meta-Analyses” (PRISMA) [27], which involved the steps of identification, screening/elimination, eligibility, and inclusion; they are briefly described below.

### 2.2. Literature Identification

The Web of Science database was used for the literature survey since its start in 1945 extending until 2019. The search was carried out in two phases, the first of which used general keywords for a broad initial search encompassing the biopesticide-related terms: “biorational”, “bioinsecticide”, “botanical”, “extract”, “phytoinsecticide”, “plant or essential-oil”, “microbial”, “mineral”, “growth-inhibitor”, “growth-regulator”, and “*Bacillus thuringiensis*”, in mandatory combination with terms “insecticide” and “insect”. This search recognized a total of 15,490 scientific manuscripts. This initial dataset was used for further (title and abstract) screening in a subsequent phase to recognize the papers focusing on plant-derived or botanical insecticides.

### 2.3. Literature Screening and Elimination

The manuscripts identified as described above were further screened by checking the relevance and consistency with the research objective here established. Thus, the adherence to the following criteria was emphasized: (1) research article published in a peer-reviewed journal; (2) report of a botanical insecticide tested against a target and/or non-target organism; and (3) description of the response assessed in target and/or non-target organisms. In contrast, the criteria for exclusion included reviews, books, theses, dissertations, and publications from meetings or conferences.

### 2.4. Literature Eligibility and Inclusion for Meta-Analysis

The selected manuscripts were further scrutinized, and their information compiled and summarized (Table S1). This dataset was used for the building of two interaction matrixes, which identified the relationship among botanical families and arthropod orders tested to recognize the botanical family diversity used as plant-derived insecticides. Thereafter, we added another eligibility criterion for inclusion in the meta-analyses, which was the use of at least one of the main plant families recognized as prevalent among the surveyed studies.

### 2.5. Statistical Analyses

A meta-analysis with binary outcomes was used to test the prevailing odds of non-target organism testing and the type of response assessed. Within such context, the risk ratio and 95% confidence intervals were used to determine the overall effect measured, where the former (i.e., risk ratio, RR) is the likelihood of an outcome between two alternative ones (RR = 1 means a similar outcome among two possibilities, or lack of bias). These estimates were subjected to formal meta-analyses with a random-effect model considering plant family and type of insect response assessed as moderators. The random-effect model was used because the individual studies differ, and their effects are heterogeneous. The quantification and heterogeneity-test (i.e., Q, H, and I^2^) were conducted, and the inverse variance and DerSimonian–Laird methods were used to estimate the between-study variance (τ^2^). Studies with n ≤ 1 event in both groups were excluded from the meta-analyses. All analyses were performed using the R-software version. 3.5.1 (R Development Core, Vienna, Austria), with the packages “meta”, “metafor" and “stats” [28,29]. The graphical illustrations were produced with Wacom creative table (Intuos S, Tokyo, Japan) using Corel Painter (Essential 6, Ottawa, ON, Canada).

## 3. Results

### 3.1. Literature Survey Summary

**Figure 1:**
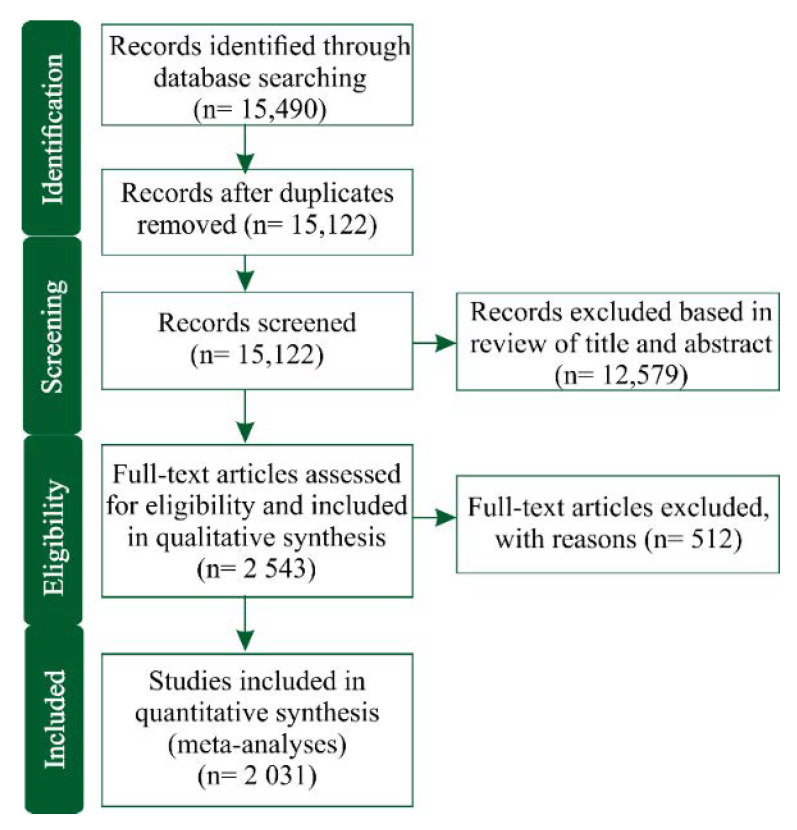
Flowchart diagram describing how scientific articles were gradually included/excluded in the literature dataset at the four stages of the systematic review process (“identification”, “screening”, “eligibility”, and “included”) for subsequent meta-analyses.

The initial literature search in the Web of Science database resulted in 15,490 papers, but after duplicate removal, the number reduced to 15,122 papers, which were subsequently screened based on titles and abstracts. Of these papers screened, 2543 papers met our initial selection criteria and were suitable for the purposes of the qualitative analyses desired. A summary flowchart of the article selection procedure following the PRISMA guidelines is exhibited in Figure 1 and summarized in a supplementary spreadsheet (Table S2).

### 3.2. Qualitative Overview and Temporal Trends

**Figure 2:**
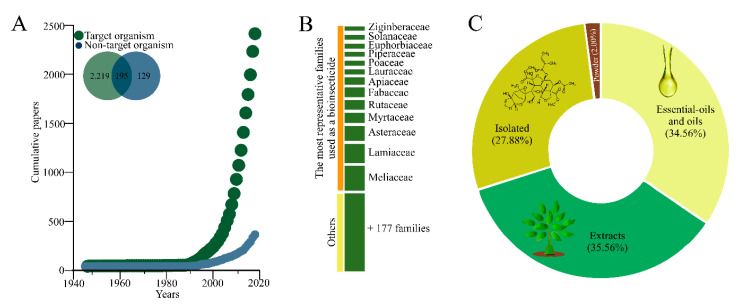
(**A**) Scatterplot of the cumulative number of manuscripts with botanical insecticides published between 1945–2019; (**B**) major plant families explored searching for botanical insecticides; (**C**) and type of formulation/composition used in studies with botanicals insecticides.

The studies with botanical insecticides started by the mid-1940s, but experienced exponential growth after the 1990s, trend which is currently maintained (Figure 2A). Target pest species received most of the attention among the identified studies with non-target arthropod species receiving attention on only 12.7% of the studies, while 7.7% of the studies assessed both target and non-target arthropod species. A large number of family plant species were explored, some 190, but 13 of them drew the bulk of attention (Figure 2B). These studies largely used either essential oils, raw extracts, and isolated compounds as source of insecticidal activity with a small proportion using plant powder (2.00%) (Figure 2C).

**Figure 3:**
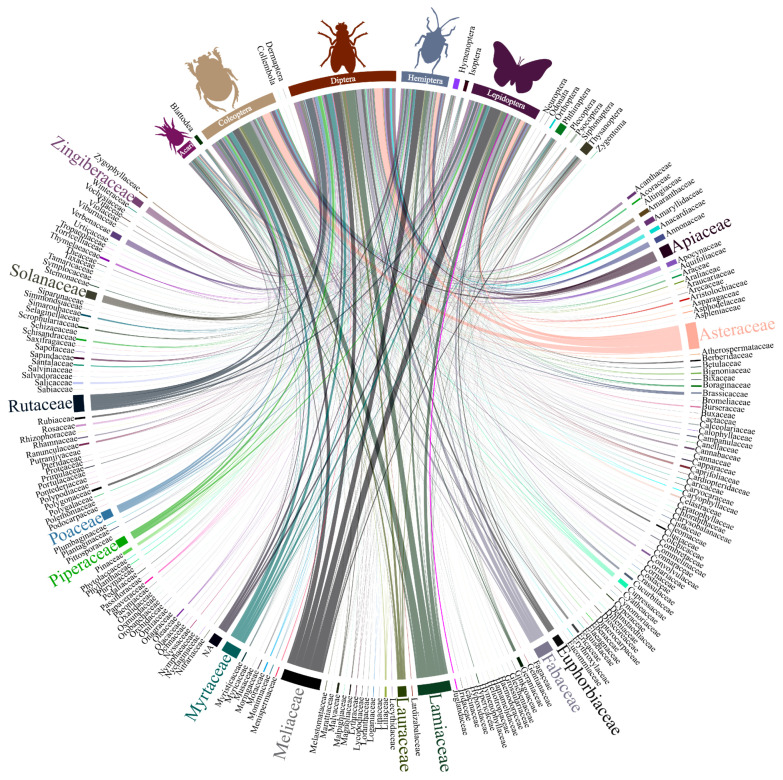
Interaction among the diversity of plant families and target organism from the literature survey of papers on botanical insecticides (n = 2543); the bar and line thickness under each taxon or connecting them corresponds to the relative number of papers dealing with the said taxa.

The research on botanical insecticides primarily involved half a dozen plant families (Meliaceae, Lamiaceae, Asteraceae, Myrtaceae, Rutaceae, and Fabaceae) and were oriented mainly towards arthropod pest species as target organisms with emphasis on the beetles, flies and mosquitos, caterpillars, hemipterans, and mites (Figure 3).

**Figure 4:**
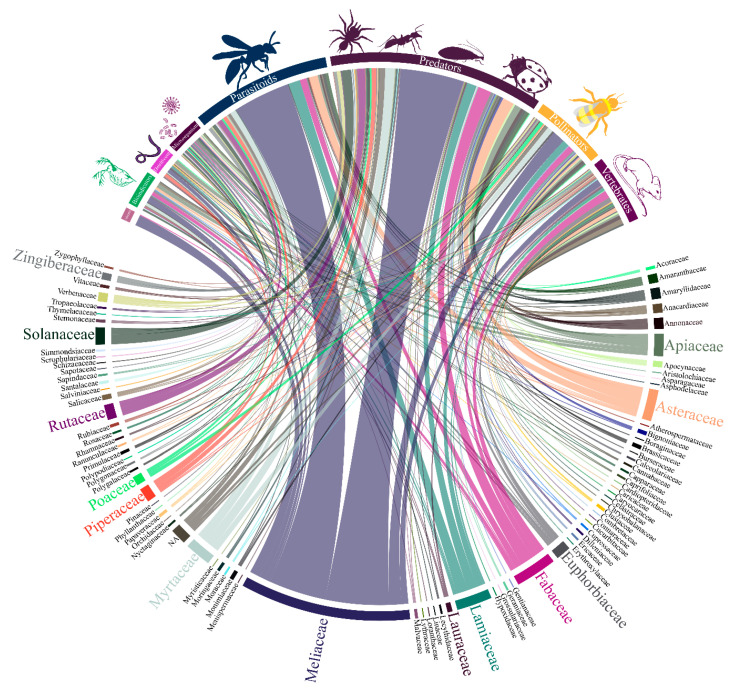
Interaction among the diversity of plant families and non-target organism from the literature survey of papers on botanical insecticides (n = 2543); the bar and line thickness under each taxon/group or connecting them corresponds to the relative number of papers dealing with the said taxa and group of non-target organisms.

The same main plant families were the focus of attention when non-target species were considered in studies with botanical insecticides, where predators, followed by parasitoids and pollinators among arthropods, besides vertebrates, received the highest share of attention (Figure 4).

### 3.3. Meta-Analyses: Quantitative Overall Trends

**Figure 5:**
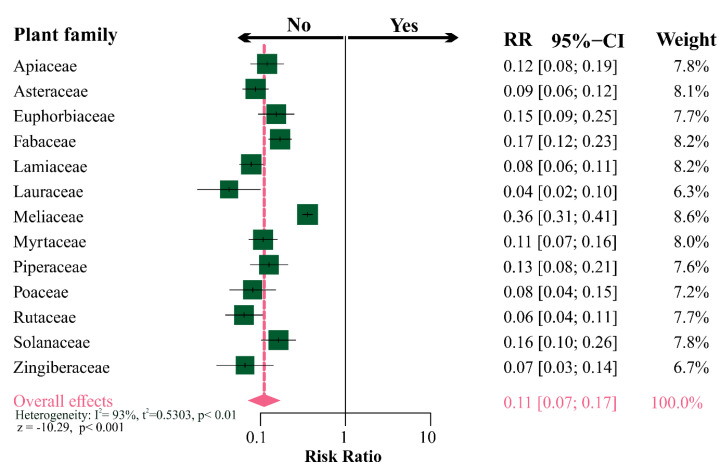
Forest plot summarizing the results from a meta-analysis on the incidence of studies on non-target organisms subjected to botanical insecticides from different plant families. The risk ratio by plant family (95% CIs) is indicated by dark green squares (horizontal black lines). The combined risk ratio estimate for all botanical families is represented by a pink diamond, where diamond width corresponds to 95% CI bounds. The vertical full-line represents lack of effect or bias (RR = 1). The *P*-values for the random-model and heterogeneity test of risk ratio by plant family are indicated.

The incidence of studies on botanical insecticides from the most investigated plant families on non-target organisms was tested to detect if the perceived bias against the latter was restricted to some plant species or was really general trend. The overall effect estimated by the random meta-analysis model indicated that it is about 10× lower; thus, the low likelihood of studies testing the effects of botanical insecticides in non-target organisms (i.e., risk ration = 0.11; *z* = −10.29; *p* < 0.001). However, it is noteworthy that the dataset exhibited a high heterogeneity among plant families (I^2^ = 93.0%, *p* < 0.01), where Meliaceae were more frequently tested against non-target organisms when contrasted to other plant families. However, even so, it is evident that there is a bias against studies with the non-target organisms in the literature (Figure 5).

**Figure 6:**
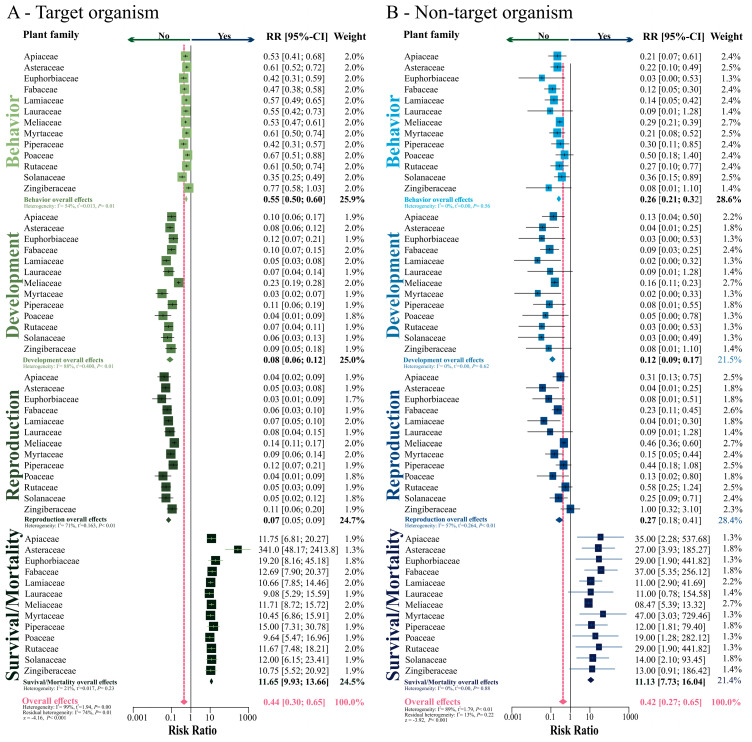
Risk ratio (or probability) of a biological parameter as survival/mortality, development, reproduction and/or behavior in (**A**) target organism, and in (**B**) non-target organism tested experimentally in the different families of plants.

Additional meta-analyses were performed to recognize whether behavioral, developmental, reproduction, and survival/mortality parameters have been recorded (or not) in papers with botanical insecticides. The overall trends estimated by random model in meta-analyses (see pink diamond) were similar for target and non-target organisms, which indicated the lack of papers exploring these parameters for both target (RR = 0.44; *z* = −4.16; *p* < 0.001; Figure 6A) and non-target organisms (RR = 0.42; *z* = −3.92; *p* < 0.001; Figure 6B), regardless of the plant family used as source of the insecticide. The heterogeneity in the dataset was significant for both target (I^2^ = 99%; *p* = 0.001) and non-target (I^2^ = 89%; *p* < 0.01) organisms. Such outcomes suggest that at least one group of response variables (i.e., parameters) is diverging from the overall trend detected in the meta-analyses. Indeed, survival/mortality is well-explored among the published papers, while the other parameters are relatively neglected.

## 4. Discussion

The boom in research on biopesticides and particularly botanical insecticides has been taking place as a response to environmental and safety concerns with conventional insecticides among the general public in different parts of the globe. As perceptions and misconceptions go hand-in-hand in determining public opinion with reflections on legislation and research support, the recognition of the current status, bias, and neglected opportunities within such topics deserves attention. Thus, the data-gathering and hypothesis-testing efforts considering the scope of knowledge published on the subject is amply justified.

Qualitative studies and surveys on botanical insecticides do exist, but quantitative hypothesis-driven studies on the subject remain lacking. Therefore, the systematic literature survey and meta-analyses carried out on the gathered data and reported here aimed at (i) recognizing the magnitude of evolution and focus of the studies on botanical insecticides, (ii) identifying existing bias on such studies, and (iii) realizing existing knowledge gaps on the subject that are worth pursuing. Our initial expectation based on previous studies and reviews was of increased interest on the subject, and likely an enhanced one despite some controversies [8,18]. The existence of bias among the studies was also expected, as also takes place among conventional insecticides [6,11], although already recognized and experiencing a shift in attention in recent years. Such biases likely hide knowledge gaps that deserve attention, which we also suspected. The gathered data and performed analyses largely support such expectations.

The perceived association between agriculture and the use of botanical insecticides likely took place only after some 4000 years of the onset of the former [26]. Early historical records indicate that the use of (aromatic) plant-derived insecticides as repellents against insects [30] was a prevailing domestic rather than agriculture scenario. Records of prominent agriculture use of botanicals date from Ancient Rome aiming granary protection; therefore, later than the children delousing with grounded chrysanthemum (pyrethrum) flowers during the reign of the Persian King Xerxes in 400 B.C. [30]. Regardless, since the 19th century the interest on botanical insecticides is on the increase and such interest boomed by the late 20th century extending to the present day [18,31]. In fact, the interest extended to biopesticides in general [8], but botanical insecticides represent nearly half of the studies on bioinsecticides justifying the attention on this particular set of compounds. The rate of research interest on botanicals even surpassed past expectation [18], and our own, with an exponential growth taking place since the early 1990s.

Pyrethrins and azadirachtin, respectively obtained from the chrysanthemum *Tanacetum cinerariifolium* (Asteraceae) and the neem *Azadirachta indica* (Meliaceae), remain in the forefront of commercial use among botanical insecticides and with a large share of the studies [18,20,31]. Nonetheless, the diversity of plant species and families scrutinized as source of insecticides grew even larger trespassing the 190-family marker. Despite these numbers, some 13 plant families received the bulk of attention and half dozen—Meliaceae, Lamiaceae, Asteraceae, Myrtaceae, Rutaceae, and Fabaceae—have been the focus of interest. This trend is likely due to two main reasons, the emphasis on the screening of botanical insecticides rather than the viabilization of their practical use, and the convergence of studies from a large screening basis towards a restricted set of promising families (and species, considering chrysanthemum and neem) [18,31]. The generally low potency of botanical insecticides on the source plant species, the variability in their content within the same species, the relatively reduced amount on the source material, and the common synergistic or potentiation activity of other mixed compounds all compromise the likelihood of a broader spectrum of plant species and botanical candidates for commercial use. Nonetheless, such diversity of screened plants is still relevant as model source for the prospection and development of plant-inspired (conventional) insecticides.

More recent screening studies exploring the biological activity of botanical insecticides usually include chemical characterization of the plant extract, valid for extracts and essential oils, and use of isolated compounds, besides the use of both negative and positive controls. These traits decreased the vulnerabilities of these studies, which were earlier detected [18], and subsequently mitigated both by researchers and editorial boards of scientific journals, which started explicitly requiring these study components. However, the studies with botanical insecticides still largely focus on the detection of mortality-based activity on target pest species. Mortality is an important toxicological endpoint, but the more promising use of botanical insecticides may lie elsewhere—sparking repellence or other important sublethal responses [6,11,32,33,34], what remains neglected constituting another shortcoming for the development of these compounds for commercial use.

The bias on focusing on mortality as toxicological endpoint for assessing insecticide activity is not exclusive of botanical insecticides, as here reported, or even biopesticides as a whole [8], but it is also frequent in studies with conventional insecticides. However, this trend is shifting for the latter [6,11,32], but needs also to change in studies with botanical insecticides. This is so, not only because a varied set of responses can potentially lead to suppression of pest populations and the injury they cause, but because sublethal exposure prevails in the field due to environmental degradation of these chemicals extending the length of this (sublethal) exposure to much longer periods than the lethal exposure, and because non-target species are also present in the system and are usually sublethally affected by these insecticides [6,8,11].

The lethality bias and the focus on control of pest species, together with the general perception and assumption of higher environmental safety levels associated with botanical insecticides, probably discourages the testing of non-target organisms, another detected bias on studies with botanical insecticides. This is a serious misconception with consequential shortcomings both for their commercial use for pest management and regulatory considerations. Lack of selectivity is a problem detected for azadirachtin against natural enemies and pollinators compromising their potential use at least in some circumstances [6,23,33,34,35], but it is unlikely to be restricted only to this biorational. In addition, resistance to botanical insecticides, although potentially more difficult to take place, does occur even under sublethal selection, as long as the selection pressure is enough for the differential survival and reproduction of the resistant individuals [8,11].

The regulatory scenario also suffers from the reported bias of studies with botanical insecticides. Improved formulation needs and higher manufacturing costs are broadly recognized as drawbacks for developing commercial botanical insecticides [13]. However, presumed safety for humans and environment should not be taken for granted by regulatory bodies and the policies regarding that greatly vary from country to country [8,13,31]. Lack of studies on non-target organisms is a problem, as already mentioned, and among those, humans should not be neglected either. One just needs to remind of the association between rotenone and human neurodegenerative diseases [36], and the high acute toxicity of nicotine and strychnine [37], to recognize this fact. Nonetheless, it is always important to emphasize that the origin of an insecticide, whether synthetic or natural (vegetal, animal, or microbial), is not a determinant of toxicity or safety, which is a function of the compound structure and its associated physical-chemical properties. This fact is well-known, but paradoxically neglected in diverse arenas, including the public one. Thus, sobering attention is necessary for credible and sustainable use of botanicals, and of any other (bio)pesticide for that matter.

## 5. Conclusions

Our literature survey and analyses of studies on botanical insecticides spanning 74 years (1945–2019) recognized the exponential growth of such studies and the primary focus on about a dozen family species as source of such compounds. In addition, two prominent biases were detected in these studies—the broad focus on pest species, neglecting non-target organisms, and the primary focus on mortality estimates, neglecting sublethal assessments. Both biases are consequential to pest management and regulatory agencies reverting into knowledge gaps worthy of attention if credible and sustainable use of such compounds is the objective.

## Supplementary Materials

The following is available online at https://www.mdpi.com/2075-4450/11/8/532/s1, Table S1: Operational questions to systematize the survey of publications on the use of botanical insecticides, Table S2: Listed of retrieved articles and their respective summaries that were used for survey and meta-analyses.
