# Supplementary material for: Plant-Derived Insecticides Under Meta-Analyses: Status, Biases, and Knowledge Gaps
Source: Insects. 2020 Aug 14;11(8):532. doi: 10.3390/insects11080532 (PMC7469165; doi:10.3390/insects11080532)
Supplement: Supplementary file 1 [file insects-11-00532-s001.zip › Table S1.docx]

**Table S1.** Operational questions to systematize the survey of publications on the use of botanical insecticides.

| **Questions** | **Description** |
| --- | --- |
| What is the type of “formulation or botanical insecticide” used in the paper? ^a^ | We identified the type of “formulation or botanical insecticide” according to the following subcategories:   1. **Plant powder**: when plant parts (e.g., leaves, trunk, roots, rhizomes, flowers, and fruits) were ground to produce powder, which was used directly or diluted in a carrier such as clay, talc, or even with diatomaceous earth. 2. **Plant extracts**: when used a crude preparation from the powder of plant parts that were posteriorly added an organic solvent (e.g., water, acetone, and alcohol), as well as the separation of extract at different enriched fractions. 3. **Plant essential-oil**: when used a substance which is obtained by steam distillation with or without the addition of water to the still. 4. **Molecules isolate from plants**: when used a molecule present in the plants, which could be isolated from them (i.e., a substance with a higher purity degree) or artificially synthesized. |
| What is the plant family (taxonomic rank) studied? | All the plants were listed and identified according to the taxonomic family. In order to standardize the nomenclature, we used the website (http://powo.science.kew.org/) a project from Royal Botanic Gardens, which provides the current nomenclature from plant species. **NA** was used when the manuscript did not contain a family describes. |
| What is the target-organism group (taxonomic rank) studied? ^b^ | All target organisms of insecticides (i.e., majority insect and acari) were listed and identified according to the taxonomic order. **NA** was used when the manuscript did not contain a target organism. |
| What are the non-target groups studied? ^b^ | We identified the non-target organism group according to the following subgroups:   1. **Abiotic**: which including the studies that explore the persistence from botanical insecticides in the environment. 2. **Bioindicators**: which including the studies with the living organism that gives us an idea of the health of an ecosystem (e.g., Microcrustacean and Ephemeroptera) 3. **Detritivores**: which including the studies with a heterotrophic organism, which obtains its nutrition by feeding on detritus (e.g., Earthworms) 4. **Microorganism:** which including the studies with protozoa, bacteria, and/or fungi that did not exhibit a clear ecological function. 5. **Parasitoids**: which including the studies with organisms that its progeny develop on or within another organism (the host), eventually killing it (e.g., Hymenopterans). 6. **Predators**: which including the studies with organisms that capture and eats another (the prey) (e.g., coleopterans, hemipterans, acari, fish, and spiders). 7. **Pollinators**: which including the studies with organisms that move pollen from anther to the stigma of a flower (e.g., majority bees). 8. **Vertebrates**: which including the conducted with fishes, amphibians, reptiles, birds, and/or mammals, which was not included in some subgroup listed above. 9. **NA**: was used when the manuscript did not contain a non-target organism. |

^a^ These categories are not mutually exclusives by papers; ^b^ A paper may contain more than one plant family.
